# Children’s shoe styles and parent decisions to fit shoes with store staff assistance

**DOI:** 10.1186/1757-1146-4-S1-O35

**Published:** 2011-05-20

**Authors:** Stefania Penkala, Lynne Harris, Adrienne Hunt, Geraldine Naughton

**Affiliations:** 1 Biomedical and Health Sciences, University of Western Sydney, Locked bag 1797 Penrith South DC, NSW, Australia; 2 Health Sciences, University of Sydney, Lidcombe, NSW, Australia; 3 Exercise Sciences, Australian Catholic University, Strathfield, NSW, Australia

## Background

A well-fitted shoe is singularly the most consistent footwear attribute recommended in children’s footwear guidelines to promote foot health. Despite recommendations research over the last 70 years continues to report the majority of children wear inappropriately sized shoes. It would be beneficial for the development of consumer appropriate guidelines to know what influences parents when making decisions about the different types of shoes they purchase for their child. The aim of the study was to obtain contemporary information about decisions made by parents to fit their child’s shoes and the fitting problems they experience.

## Methods

Parents with children aged 4 to 12 years (n=272) were recruited in Sydney using a multistage cluster stratified sampling procedure. A self-administered 156 item survey was distributed once via the child’s school. Shoe type and fitting practices are described here. Descriptive and Pearson chi square analysis were used to describe the data and detect associations between categorical data. The study received ethics approval from the University of Sydney Human Research Ethics Committee and the NSW Department of Education and was supported by Clarks Australia.

## Results

More parents sought professional assistance from store staff to fit their child’s school shoes (64%), than physical activity shoes (59%) and casual shoes (44%). A small number of parents estimated shoe size without their child present for school (4%), physical activity (5%) and casual wear (7%). For school shoes, traditional lace-up and Mary-Jane/ high-heel shoes were more likely to be fitted with assistance than athletic style shoes (χ^2^(2) = 8.34; p = 0.02). For both physical activity (χ^2^(2) = 9.10; p = 0.01) and casual shoes (χ^2^(2) = 10.20; p = 0.006), sandal style shoes were the least likely to fitted with assistance compared to athletic style shoes. Difficulty finding shoes that fitted their child’s foot was reported by 36% of parents. School shoes were more likely to be fitted with assistance when parents found it difficult to find shoes that fitted their child’s foot (χ^2^(2) = 7.63; p = 0.02). The associations between ease of fit and fitting assistance were not significant for physical activity (χ^2^(2) = 1.84; p = 0.40) or casual shoes (χ^2^(2) = 0.63; p = 0.43).

## Conclusions

Shoe-fitting practices were affected by the shoe style purchased. While parental shoe practices vary, most parents prioritize assisted shoe-fitting for school and physical activity shoes. Among school shoes, athletic style school shoes were the least likely to be fitted with assistance, as were sandals for casual wear. The poor availability in size ranges for athletic and casual shoe styles, may explain why parents did not seek assistance when fitting these shoes styles. However, given prioritizes to encourage physical activity in and out of school, and the importance of shoe comfort for foot health, educational strategies are needed to ensure parents have the skills and resources to monitor shoe fit.

